# Advancements in Cell-Based Therapies for HIV Cure

**DOI:** 10.3390/cells13010064

**Published:** 2023-12-28

**Authors:** Yusuke Matsui, Yasuo Miura

**Affiliations:** 1 Gladstone Institute of Virology, Gladstone Institutes, 1650 Owens St., San Francisco, CA 941578, USA; 2 Department of Transfusion Medicine and Cell Therapy, Fujita Health University School of Medicine, 1-98 Dengakugakubo, Kutsukake, Toyoake 470-1192, Aichi, Japan

**Keywords:** HIV-1, AIDS, CAR-T cell, CD34^+^ cell, CD4^+^ T cell, CRISPR/Cas9, clinical trials, latency, latency-reversing agents, combination ART, cure study, cytotoxic T lymphocyte, CCR5, functional cure, elite controller, Berlin Patient, cytokine release syndrome, off-the-shelf

## Abstract

The treatment of human immunodeficiency virus (HIV-1) has evolved since the establishment of combination antiretroviral therapy (ART) in the 1990s, providing HIV-infected individuals with approaches that suppress viral replication, prevent acquired immunodeficiency syndrome (AIDS) throughout their lifetime with continuous therapy, and halt HIV transmission. However, despite the success of these regimens, the global HIV epidemic persists, prompting a comprehensive exploration of potential strategies for an HIV cure. Here, we offer a consolidated overview of cell-based therapies for HIV-1, focusing on CAR-T cell approaches, gene editing, and immune modulation. Persistent challenges, including CAR-T cell susceptibility to HIV infection, stability, and viral reservoir control, underscore the need for continued research. This review synthesizes current knowledge, highlighting the potential of cellular therapies to address persistent challenges in the pursuit of an HIV cure.

## 1. Introduction

The emergence of combination antiretroviral therapy (ART) in the 1990s represented a pivotal milestone in the management of human immunodeficiency virus (HIV) infection, fundamentally transforming the treatment landscape [1]. Since this breakthrough, there has been an unwavering effort to refine ART regimens, with a specific emphasis on enhancing safety, tolerability, efficacy, and overall ease of use. Presently, single-tablet regimens [2] (daily tablets containing from two (e.g., dolutegravir/lamivudine) to three drugs (e.g., bictegravir/emtricitabine/tenofovir alafenamide)) or monthly injections (cabotegravir and rilpivirine [3]) provide sustained viral suppression for adherent patients. This suppression can prevent transmission (the concept of “Undetectable = Untransmittable, U = U” [4,5]). Additionally, prophylactic measures, including daily pills or bi-monthly injections (cabotegravir [6,7]), have proven effective in preventing HIV infection among individuals at high risk.

However, despite these notable advancements, the HIV epidemic endures globally, resulting in 1.3 million new infections and approximately 630,000 HIV-related deaths each year [8]. The Joint United Nations Programme on HIV/AIDS (UNAIDS) Fast-Track Initiative is geared towards reducing new infections to fewer than 200,000 annually, aiming to eradicate stigma through the “95-95-95” strategy, in which 95% of those infected with HIV are aware of their status, 95% receive treatment, and 95% achieve viral load control below detection sensitivity. Realizing this objective requires widespread and continuous testing and treatment to maintain viral suppression across communities. Despite the anticipation of longer-acting ART [9,10], the current ART-focused response encounters challenges, including susceptibility to fluctuations in public health funding. Indeed, as of 2022, an estimated 24% of the world’s HIV-infected individuals were not receiving antiviral treatment [8]. Long-term ART may also give rise to complications stemming from chronic, persistent inflammatory conditions [11,12], alongside potential challenges related to stigma. For these reasons, research endeavors worldwide are underway to pursue an HIV cure, aiming to eliminate all replication-competent HIV viruses from infected individuals or to achieve control over viral replication from the HIV reservoir [11] without the need for ongoing antiretroviral therapy (functional cure).

Throughout the HIV life cycle, the provirus becomes integrated into the host genome. While the majority of the integrated viral genome is defective, a small fraction of cells infected with intact viral genomes (typically <5–10% in circulating HIV-1-infected CD4^+^ T cells) transition into a state of HIV latency [12,13,14]. These provirus-bearing cells serve as HIV reservoirs that are capable of transcribing the provirus and releasing new virions upon interruption of ART. Various approaches have been explored to achieve the long-term neutralization of or reduction in the latent HIV reservoir. Among these, the “shock-and-kill” strategy garnered initial attention. This concept suggests that inducing HIV provirus transcription with latency-reversing agents (LRAs) could result in the killing of infected cells through viral activity or via the host’s immune response. However, such interventions have not yet been shown to diminish the reservoir size in in vivo studies [15]. Another strategy, the “block-and-lock” approach, involves the permanent silencing of integrated provirus. To date, numerous compounds known as latency-promoting agents (LPAs), which hinder viral transcription, have been identified as candidates and are currently undergoing preclinical trials [16]. Alternatively, some researchers have sought to directly eliminate proviruses through gene editing. In animal models, the administration of CRISPR/Cas-based enzymes designed to excise substantial segments of the HIV provirus, facilitated by adeno-associated virus 9 (AAV-9), has demonstrated efficacy [17]. This approach is presently undergoing clinical trials (NCT05144386) in human subjects [18].

In addition to targeting infected cells directly, methods focusing on the immune system are currently under development. Broadly, these immunotherapy approaches seek to augment the proficiency of endogenous T cells in recognizing HIV (e.g., with vaccines [19]) or to engineer T cells with improved cytotoxic capacity. Indeed, in investigations involving elite controllers, individuals capable of prolonged HIV control without resorting to ART, it has been observed that viral control is predominantly mediated by CD8^+^ T-cell responses [20,21,22,23], indicating the potential of these cells as therapeutic tools. In this review, we provide a comprehensive summary of ongoing trials aimed at developing cell-based therapies for achieving an HIV cure, with a particular emphasis on the utilization of CAR-T cell therapies.

## 2. Attempts to Functionally Cure HIV Using Cell-Based Therapies (Cytotoxic Approach)

### 2.1. Investigation of CAR-T Cell Structures

**Figure 1:**
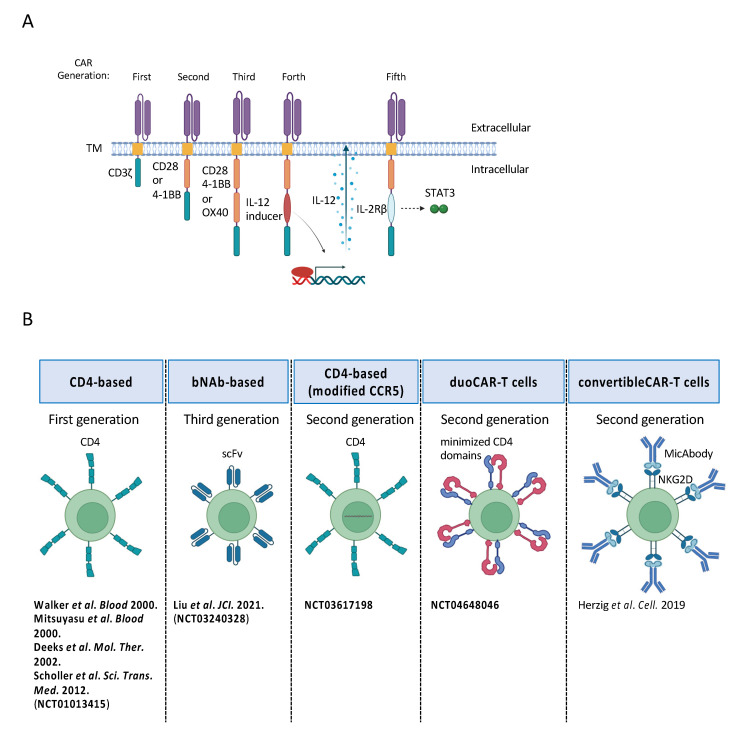
(**A**) Schematic of the structure of a chimeric antigen receptor (CAR) [33,34,35]. The first generation incorporates the CD3ζ chain as the intracellular signaling component. In the second generation, additional co-stimulatory molecules, such as CD28 [36] and 4-1BB [37], are introduced. The third generation is characterized by the presence of two co-stimulatory molecules aimed at enhancing cytotoxicity and promoting long-term viability. In the fourth generation [33,40], an intracellular interleukin domain, IL-12, is integrated, leading to the release of IL-12 upon CAR expression. The fifth generation introduces a cleavage domain of IL2Rβ and a STAT3-binding motif to the intracellular domains of CD28 and CD3ζ, further augmenting proliferation and ensuring prolonged viability. (**B**) Research on CAR-T cells for HIV-1 infection [41,42,43,44,45,46,47]. One approach to prevent the infection of CAR-T cells themselves involves modifying the extracellular domain of the CAR, shifting from CD4 to broadly neutralizing antibodies (bNAb) [45]. Another is the genetic modification of C-C-chemokine receptor-5 (*CCR5*) to enhance resistance. Additionally, efforts are underway to employ an extracellular domain capable of recognizing multiple epitopes, serving as a countermeasure against HIV immune evasion [46]. Studies highlighted in boldface type denote clinical investigations. TM, transmembrane; scFv, single-chain variable fragments.

Among the potential cell-based therapies for HIV infection, CAR-T cell technology stands out given its established clinical success in the treatment of hematologic malignancies. The U.S. Food and Drug Administration (FDA) has approved CD19-targeted CAR-T cells for treating various types of B-cell lymphomas, including relapse/refractory aggressive [24,25,26], mantle cell [27], and indolent lymphomas [28,29]. In clinical trials, these cells have achieved complete response rates ranging from 40–74%, depending on the type of lymphoma. Notably, some patients have experienced durable responses [29,30], highlighting the potential of these cells for curing specific cases of B-cell lymphoma. Additionally, the FDA has approved B cell maturation antigen (BCMA)-targeted CAR-T cells for relapsed/refractory multiple myeloma, which have shown overall response rates of 73–98% [31,32]. CAR-T cell therapies are made by collecting T cells from a patient and re-engineering them to produce proteins on their surface called chimeric antigen receptors (CARs) that recognize and bind to specific antigens. CARs comprise three domains: an extracellular domain designed to specifically bind to the antigen, a transmembrane segment anchoring the receptor, and an intracellular domain responsible for signal transduction. CAR structures have undergone multiple modifications and presently can be categorized into five generations (Figure 1A) [33,34,35]. The first generation incorporates the ζ chain as the signaling structure. In the second generation, additional co-stimulatory molecules like CD28 [36], 4-1BB (CD137) [37], or OX-40 (CD134) [38] are introduced to the signaling domain, enhancing effector cell proliferation, persistence, cytotoxicity, and sustained response. The third generation features two co-stimulatory molecules, such as CD28 and 4-1BB, aiming to improve cytotoxicity and long-term viability. This structure promotes T cell activation and the elimination of antigen-negative target cells in the target lesion. The fourth generation of CAR-T cells, referred to as T cells redirected for universal cytokine-mediated killing (TRUCK) [39], incorporates the intracellular interleukin 12 (IL-12) domain into the CAR cassette. This design facilitates the release of IL-12 upon CAR expression, promoting T cell activation and the elimination of antigen-negative target cells within the target lesion. The fifth generation [33,40] involves the addition of IL2Rβ cleavage domains and STAT3-binding motifs to the intracellular domains of CD28 and CD3ζ to further enhance the proliferation and long-term viability of the CAR-T cells. The FDA-approved CAR-T cell therapies utilize a second-generation framework. This framework includes an antigen-binding domain, a transmembrane domain, a co-stimulatory domain (derived from either CD28 or 4-1BB), and a CD3ζ-based T cell signaling domain. Iterations on these CAR structures have been used in efforts to develop T cells that can recognize HIV-infected cells.

### 2.2. Clinical Trials of CAR-T-Cell-Based Therapy for HIV Infection

In the cytotoxic approach to combat HIV infection, CAR-T cells are designed to recognize HIV envelope proteins on the surface of infected cells, eliciting cell-killing effects (Figure 1B). CD4 is a cell surface receptor that serves as the primary receptor for HIV entry into host cells. The virus binds to CD4 on the surface of helper T cells, leading to the initiation of the viral life cycle. By engineering CAR-T cells to express a CD4-derived CAR, these modified T cells can be designed to specifically recognize and target cells infected with HIV. This targeted approach aims to enhance the specificity of the immune response, potentially reducing off-target effects and minimizing damage to uninfected cells. In 2000, Walker et al. conducted a study involving the adoptive transfer of CD4-CAR-T cells (CD4/CD3-ζ chain). The study not only demonstrated the safety and feasibility of CD4-CAR-T cells but also revealed an interesting observation: the gene-modified CD4^+^ T cells positively influenced the survival of CD8^+^ T cells. This finding suggests a potential synergistic interaction between CD4-CAR-T cells and CD8^+^ T cells, highlighting the complex and interconnected nature of immune responses in the context of adoptive cell therapy for HIV [41]. Mitsuyasu et al. also conducted a clinical study of CAR-T cells in 24 HIV-infected patients using CD4ζ gene-modified CD4^+^ T cells and CD8^+^ T cells and showed that cells expressing the HIV envelope protein gp120 were specifically cytolytic [42]. However, while this type of CAR-T cell achieved at least a 14-day reduction in HIV RNA in rectal tissue, they did not elicit a change in plasma HIV RNA. These clinical trials demonstrated the safety and feasibility of CD4-CAR-T-cell therapy, but they also indicated that much remained to be learned about how to optimize this approach.

In 2002, Deeks et al. conducted a randomized phase 2 trial comparing CD4ζ gene-modified versus unmodified T cells in 40 HIV-infected patients on ART with a plasma viral load <50 copies/mL [43]. The study observed no significant alteration in reservoir size following the administration of the gene-modified T cells. However, the infusion of gene-modified T cells was associated with a trend toward reduced viremia from baseline in two out of four reservoir assays and fewer reports of recurrent viremia. In 2012, Scholler et al. reported the outcomes of a long-term follow-up involving three clinical trials (NCT01013415). CD4ζ gene-modified T cells were identified in 98% of the samples tested, persisting for at least 11 years after injection [44]. Remarkably, the CD4ζ transgene exhibited continued expression and function over the extended follow-up period.

A limitation of CD4-based CAR-T cells is their vulnerability to HIV infection, because CD4 serves as the ligand for gp120. As an improvement measure, researchers have tested the potential of incorporating broadly neutralizing antibodies (bNAbs) targeting various epitopes of the HIV gp160 protein. For example, Hale et al. integrated single-chain variable fragments (scFv) from four distinct antibodies (VRC07-523 [48], PGT-128 [49], PGT-145 [50], and 10E8 [51]) as antigen-recognition domains within a CAR construct. In comparison to CD4-based CAR-T cells, CAR-T cells based on bNAbs demonstrated a superior neutralizing capacity against various HIV-1 strains and exhibited resistance to HIV in vitro [52]. Furthermore, in 2021, Liu et al. conducted a clinical trial (NCT03240328) of third-generation CARs linked to the bNAb scFv and showed that the therapy eliminated reactivated latently infected cells [45]. Their findings indicated that CAR T-cell therapy did not change the time to viral rebound following ART discontinuation but did result in a significant reduction in cell-associated viral RNA and intact proviruses. Also, a clinical trial (NCT03617198) is currently in progress, utilizing second-generation CD4-CAR-T cells treated with C-C-chemokine receptor-5 (*CCR5*)-targeting zinc finger nucleases (ZFNs). CCR5, a co-receptor on the surface of CD4^+^ T cells, plays a crucial role in the entry of HIV into these cells [53]. Modification of the *CCR5* gene in CD4^+^ T cells aims to mimic the natural resistance observed in individuals with the *CCR5-*Δ32 mutation, rendering the cells less susceptible to HIV-1 entry. Thus, so far, efforts have been made to explore CAR-T cell therapy using CD4-CAR-T cells as a prototype.

### 2.3. Modification of CAR-T Cell Treatment for HIV Infection

One challenge associated with CAR-T cell therapy for HIV infection is the potential for immune escape resulting from mutations in gp120. Therefore, it is crucial to arm CD8^+^ T cells with CARs capable of recognizing a diverse range of HIV antigens. Notably, elite controllers, who exhibit superior functional activity, have demonstrated broader variant cross-reactivity in their cytotoxic T lymphocyte (CTL) response compared to non-controllers [34,35]. Efforts to circumvent HIV immune escape involve exploring duoCAR-T cells that target multiple highly conserved sites on gp160 (Figure 1B) [54,55]. Multi-site-targeting CAR-T cells, engineered to express two or three gp120/gp41 domains, are proposed to offer advantages over their single-site counterparts, potentially delaying or evading the mechanisms of HIV immune escape. A current clinical study utilizing duoCAR-T cells (NCT04648046) is actively investigating this methodology [46]. These cells target distinct gp120 epitopes through separate CARs, reducing the CD4 domain that binds to gp120 and exposing the m36 epitope (CD4-induced site [56]), which is recognized by the second CAR. The stimulatory molecule 4-1BB was also integrated into the design. This ongoing study is a dose-escalation trial, ranging from 3 × 10^5^ to 10^6^ cells per kg of body weight, with certain patients concurrently receiving cyclophosphamide to enhance the engraftment and persistence of the CAR-T cells.

Alternatively, convertible CAR-T cell technology has emerged as a strategy for developing CAR-T cells capable of recognizing multiple antigens through the use of antibodies (Figure 1B) [57,58]. In the context of HIV, convertible CAR-T cells leverage the robust binding affinity of the inactive natural killer group 2D (NKG2D) receptor ectodomain and its corresponding ligand, minor histocompatibility antigen (MICA), to engineer a dual-specificity antigen-targeting adapter molecule (MicAbody) integrated with bNAbs. The combination of multiple bNAbs and adjustable quantities of MicAbody represents a versatile approach. Notably, successful studies have reported the effective elimination of both HIV-infected CD4^+^ T cells and reactivated CD4^+^ reservoir T cells by convertible CAR-T cells in both in vitro and ex vivo settings [47].

An additional challenge associated with CAR-T cell therapy for HIV infection is the difficulty in recognizing the target cells if they are latently infected. Liu et al. reported that third-generation CAR-T cells could effectively suppress viral rebound in CD4^+^ latently infected cells that were activated with vorinostat or bryostatin1 [59]. Augmenting HIV-specific CTL responses before viral activation with LRAs may facilitate the swift elimination of infected cells. Moreover, there have been efforts to control latently infected cells by directing CAR-T cells to localize in tissues crucial for HIV reservoir formation. For example, Pampusch et al. employed CD4-CAR-T cells expressing CXCR5, guiding the cells to B cell follicles through the interaction with the chemokine CXCL13, in in vivo experiments [60]. The CAR-T cells were observed to localize to lymphoid follicles and reduce the follicular viral RNA level. Besides lymph nodes, the brain is a critical organ to consider for CAR-T cell delivery in HIV therapy. This organ contains microglia, a known target for HIV, capable of prolonged virus production [61,62]. Recent clinical trials have revealed that CD19-targeted CAR-T cells, when administered intravenously, are able to penetrate the blood–brain barrier (BBB) [63,64]. In cerebrospinal fluid (CSF), CAR-T cells have been observed to maintain high levels for a duration of at least six months [65]. However, despite this progress, there are still limited data on the efficacy of CAR-T cells in eradicating HIV reservoirs in models of HIV infection within the central nervous system. Therefore, despite ongoing efforts to develop CAR-T cell therapy for HIV-1, it remains unclear how to consistently reduce the viral reservoir at this time.

## 3. Research on Treatment of HIV Infection by Cells with Disrupted *CCR5* Gene

An additional cell-based therapy being investigated is the construction of T cells resistant to HIV infection. HIV-1 enters target cells through the CD4 receptor in conjunction with either the CCR5 co-receptor [66] or the CXCR4 co-receptor [67]. Exploring cellular therapies targeting the *CCR5* gene marks a significant advancement in HIV treatment. This gene encodes a protein essential for HIV’s entry into CD4^+^ T cells. Individuals with a natural *CCR5*-Δ32 mutation, which leads to the absence of this gene, show resistance to HIV infection. The importance of the *CCR5* gene in HIV-1 research was first highlighted by epidemiological studies, which showed that individuals with the *CCR5*-Δ32 mutation were less susceptible to HIV infection [68]. This finding spurred research into gene editing as a potential HIV cure, initially focusing on stem cell transplantation from donors with the *CCR5*-Δ32 mutation. At least five cases of cures for HIV infection have been reported after allogeneic hematopoietic stem cell transplantation from a donor homozygous for a 32-bp deletion in the *CCR5* gene [69,70,71,72,73,74]. The “Berlin Patient” [69,73], who remained cured for over a decade until his demise, underwent two bone marrow transplants in 2007 from a donor with a homozygous *CCR5* Δ32 deletion to treat acute myeloid leukemia and has not required antiretroviral therapy since then. The “London Patient” [70] received a similar transplant in 2016 for Hodgkin’s lymphoma treatment from a donor with the same genetic deletion and has been off antiretroviral therapy since 2017, with no irradiation involved in the pre-treatment. In 2013, the “Düsseldorf Patient” [71] also underwent a bone marrow transplant from a donor with a homozygous *CCR5* Δ32 deletion for acute myelogenous leukemia. He discontinued antiretroviral therapy five years later and has maintained a cured state for over two years. The “New York Patient” [72], a mixed-race woman, received a *CCR5*Δ32/Δ32 homozygous cord blood unit, combined with CD34-selected stem cells from a haploidentical related donor with *CCR5* wild-type allele, for acute myeloid leukemia treatment in 2017. She has been off antiretroviral therapy for 37 months post-transplant. Finally, the “City of Hope Patient” [74], a 63-year-old man, received a bone marrow transplant in 2019 from a donor with the homozygous *CCR5* Δ32 deletion for acute myeloid leukemia. Reduced-intensity conditioning (RIC) was chosen as the pre-transplantation treatment. These findings have catalyzed the development of a new therapeutic approach: modifying the *CCR5* gene in cells to mimic this innate resistance.

Therefore, to create immune cells resistant to HIV, scientists have tested the potential of incorporating the *CCR5* gene modification into hematopoietic stem and progenitor cells, CD4^+^ T cells, and induced pluripotent stem cells. Tebas et al. utilized a ZFN to modify the *CCR5* gene in CD4^+^ T cells, which were then injected into 12 patients [75,76]. During the interruption of ART and the subsequent increase in viral load, the decline in *CCR5*-modified cells was less pronounced compared to unmodified cells. Moreover, in most patients, blood levels of HIV DNA decreased. *CCR5*-modified CD4^+^ T cells constituted 13.9% of total CD4^+^ T cells at one week, with an estimated mean half-life of 48 weeks. Xu et al. achieved the successful transplantation and engraftment of hematopoietic stem and progenitor cells with a CRISPR/Cas9-modified *CCR5* gene in patients with both HIV infection and acute lymphoblastic leukemia [77]. The researchers isolated CD34^+^ cells from these patients and subsequently transfected them with a ribonucleoprotein complex consisting of the Cas9 protein and two guide RNAs targeting *CCR5*. The efficiency of insertion or deletion (indel) in the *CCR5* gene within CD34^+^ cells was measured at 17.8%. However, the percentage of *CCR5* disruption in CD4^+^ T cells post-transfection was approximately 2.5%. Seven months after transplantation, ART was disrupted. Unfortunately, the serum viral load increased in these patients after four weeks after ART cessation, prompting the resumption of ART. A comparable endeavor using induced pluripotent stem cells was conducted by Levy et al. [45,53]. That group derived CD34^+^ cells from induced pluripotent stem cells and genetically modified them in vitro to carry the *CCR5*Δ32 mutant allele, but the cells were found to be nonviable in humanized mice. However, the intramuscular injection of gene-edited induced pluripotent stem cells in mice resulted in the formation of teratomas, and CD34^+^ cells isolated from the teratomas demonstrated successful engraftment. Ensuring the successful in vivo engraftment of in vitro gene-edited cells presents a forthcoming challenge in the field.

## 4. The Potential Synergistic Effects of Combining *CCR5* Gene-Edited CD4^+^ T Cells with CD8^+^ CAR-T Cells

**Figure 2:**
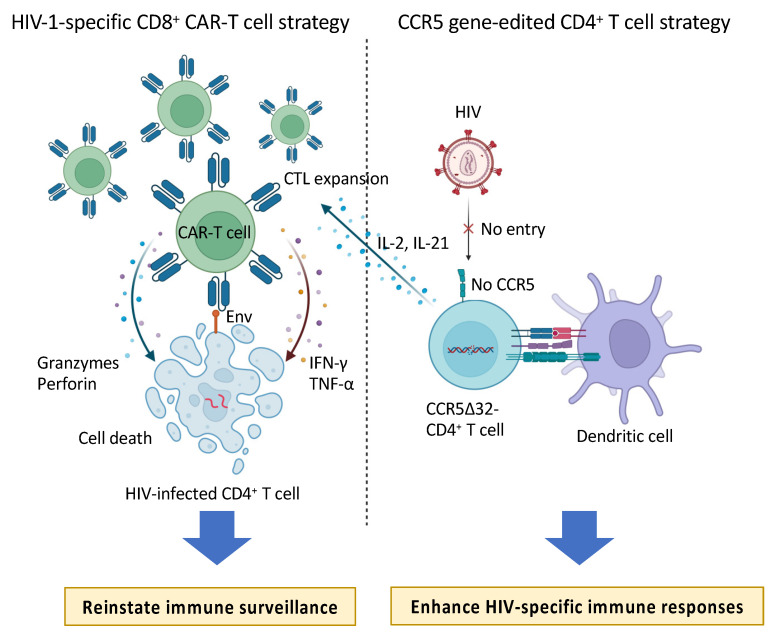
Two cellular therapeutic strategies against HIV-1. CD8^+^ CAR-T cells aim to directly reinstate cytotoxic functionality against HIV-1-infected cells by providing supplementary cytotoxic T lymphocytes (CTLs). Conversely, the strategy involving *CCR5*-modified CD4^+^ T cells focuses on activating HIV-1-specific immunity through the introduction of infection-resistant T cells. Based on the outcomes of prior studies, these two strategies appear both promising and complementary. Further investigation, including exploration of combination therapy, is warranted to optimize their potential efficacy.

The CAR-T cell-based strategy aims to reinstate immune surveillance in HIV-1-infected patients by the adoptive transfer of HIV-1-specific CD8^+^ CAR-T cells grown ex vivo, which can directly target virus-producing cells. Alternatively, the infusion of hematopoietic stem and progenitor cells or CD4^+^ T cells edited for the *CCR5* gene into HIV-1-infected patients [75,76,77] can generate CD4^+^ T cells resistant to HIV-1. Given the potential for infused *CCR5* gene-edited CD4^+^ T cells to enhance existing HIV-specific immune responses, it may be possible to synergize *CCR5* gene-edited CD4^+^ T cells with HIV-1-specific CD8^+^ CAR-T cells (Figure 2). In fact, Maldini et al. demonstrated that HIV-resistant CD4^+^ CAR-T cells (C34-CXCR4) have the capability to directly modulate HIV replication while simultaneously augmenting the immune response of CD8^+^ CAR-T cells in experiments utilizing humanized mice [78]. This combined strategy holds promise as a therapeutic modality for eradicating viral reservoirs in the clinical setting [79,80]. Furthermore, following allogeneic hematopoietic stem cell transplantation (HSCT), including from donors with the homozygous *CCR5* Δ32 deletion, in HIV-1 patients, there is a rapid depletion of the HIV-1 reservoir. Post-transplantation, HIV-specific CD8^+^ T cells initially disappear but re-emerge with weak responses several weeks later. The presence of the HIV-1 reservoir, even during complete T-cell chimerism, implies its persistence post-transplantation [81]. This observation suggests that integrating CAR-T cell therapy could be pivotal in achieving a cure for HIV.

## 5. Challenges and Considerations in CAR-T Cell Therapy

CARs have been engineered to specifically target HIV-infected cells, offering a promising avenue for treating HIV-1 infection. However, there is a risk of CARs inadvertently targeting healthy cells that express similar antigens, a phenomenon known as off-target effects [82]. Notably, studies involving CD4-CAR in HIV infection have not yet reported the cytolysis of cell lines expressing major histocompatibility complex (MHC) II, a counterpart of CD4. Upon administration, CAR-T cells are activated when they encounter their specific antigen. While they primarily target cells presenting this antigen, they may also induce a systemic inflammatory response, known as cytokine release syndrome (CRS) [83]. Symptoms of CRS, which typically emerge within 1–14 days post-infusion and can take 2–3 weeks to resolve, include high fever, hypotension, or even organ failure. The onset and duration of CRS are influenced by various factors including the CAR-T product used, the clinical trial design, the treated population, and the interventions employed. Neurotoxicity [84], another complication of CAR-T cell therapy, remains poorly understood. It is hypothesized to be related to elevated cytokine levels and the migration of CAR-T cells across the blood–brain barrier. Neurological events can occur within weeks of initiating CAR-T cell therapy, either following CRS or during ongoing treatment. To date, CAR-T therapy for HIV infection and its use in treating lymphoma in HIV-positive patients [85] have shown promising safety and efficacy. A prospective study, NCT05077527 [86], is currently in progress to assess the efficacy of axicabtagene ciloleucel [87] CAR-T therapy in treating various types of B-cell non-Hodgkin’s lymphoma in HIV-positive patients. This study focuses on the application of CAR-T therapy specifically for lymphoma in individuals who are also HIV-positive. A significant challenge of CAR-T therapy is its personalized and labor-intensive nature, coupled with high costs. In oncology, efforts are underway to develop “off-the-shelf“ [88] CAR-T therapies that can mitigate side effects, scale up production, and eliminate the need for collecting and custom-modifying T cells from each patient.

## 6. Conclusions

CAR-T cells have emerged as a cellular therapy for HIV infection, leveraging the potent functions of cytotoxic CD8^+^ T cells, as observed in studies of elite controllers and HIV vaccines. However, several challenges persist, including the need to ensure that CAR-T cells remain uninfected with HIV and remain viable in the patient’s body over an extended period. Additional concerns involve the suppression of the immune escape mechanisms of the virus and establishing control over the viral reservoir. These hurdles represent crucial areas for ongoing research and development in the field of HIV cellular therapy. However, the ever-growing knowledge of CAR-T cells emerging from the cancer field provides promise that we will overcome these challenges and position CAR-T cells as promising agents for the pursuit of an HIV cure.

## Data Availability

Not applicable.
